# Long-term health outcomes of Q-fever fatigue syndrome patients

**DOI:** 10.1017/S0950268823001401

**Published:** 2023-09-19

**Authors:** Inge Spronk, Iris M. Brus, Annemieke de Groot, Peter Tieleman, Alfons G. M. Olde Loohuis, Juanita A. Haagsma, Suzanne Polinder

**Affiliations:** 1 Department of Public Health, Erasmus MC, University Medical Center Rotterdam, Rotterdam, The Netherlands; 2 Q-support, ’s-Hertogenbosch, The Netherlands

**Keywords:** fatigue, health-related quality of life, long-term health, mental health, Q-fever, Q-fever fatigue syndrome, zoonosis

## Abstract

This study determined long-term health outcomes (≥10 years) of Q-fever fatigue syndrome (QFS). Long-term complaints, health-related quality of life (HRQL), health status, energy level, fatigue, post-exertional malaise, anxiety, and depression were assessed. Outcomes and determinants were studied for the total sample and compared among age subgroups: young (<40years), middle-aged (≥40–<65years), and older (≥65years) patients. 368 QFS patients were included. Participants reported a median number of 12.0 long-term complaints. Their HRQL (median EQ-5D-5L index: 0.63) and health status (median EQ-VAS: 50.0) were low, their level of fatigue was high, and many experienced post-exertional malaise complaints (98.9%). Young and middle-aged patients reported worse health outcomes compared with older patients, with both groups reporting a significantly worse health status, higher fatigue levels and anxiety, and more post-exertional malaise complaints and middle-aged patients having a lower HRQL and a higher depression risk. Multivariate regression analyses confirmed that older age is associated with better outcomes, except for the number of health complaints. QFS has thus a considerable impact on patients’ health more than 10 years after infection. Young and middle-aged patients experience more long-term health consequences compared with older patients. Tailored health care is recommended to provide optimalcare for each QFS patient.

## Introduction

The largest Q-fever epidemic worldwide happened in the Netherlands between 2007 and 2010 [1]. Q-fever is a zoonotic disease caused by the bacterium *Coxiella burnetii* [2]. About 40% of the infected individuals experience short-term complaints ranging from mild flu-like symptoms, such as fatigue and headache, to severe symptoms such as high fever, pneumonia, and hepatitis [3].

Acute Q-fever usually resolves within a few weeks; however, about 20% of the patients remain chronically fatigued and develop Q-fever fatigue syndrome (QFS) [4]. QFS patients experience persistent fatigue over six months, causing impaired health status and significant limitations in daily functioning [4–7]. In addition to fatigue, QFS symptoms include a wide range of health symptoms, such as headache, blurring of vision, impaired memory, sleeping problems, exacerbation of symptoms after exercise (post-exertional malaise (PEM)), and musculoskeletal pain [4–7].

Early works, which studied the impact of Q-fever on health outcomes up to 10 years after the acute Q-fever infection, showed that Q-fever, and especially QFS, has a prolonged negative impact on patients’ health status, health-related quality of life (HRQL), and well-being [4, 6, 8–10]. Besides, QFS has a negative economic impact on both patients and society [3]. Compared to the general population, QFS patients have substantially worse HRQL and experience more health complaints and increased levels of fatigue [6, 8, 10]. In addition, QFS is associated with reduced social function and labour participation and increased sick leave [3, 8, 10, 11].

The vast majority of previous studies included QFS patients as a subgroup, not specifically focusing on this important patient group, which has to deal with substantially more long-term effects compared with Q-fever patients not having QFS. Besides, the impact of Q-fever and QFS on patients’ lives has been studied up to 10 years post-infection [4, 6, 8–10]. As patients continue to suffer from the prolonged consequences, it is important to get insights into the impact of longer periods of time after the infection in this patient group. Focusing on QFS patients provides the opportunity to study subgroups and determinants of health and long-term consequences. An earlier study found some differences in HRQL among age subgroups, with younger Q-fever patients having a worse HRQL compared with older patients [8]. However, this study did not investigate this specifically for QFS patients nor study age-specific differences for other long-term outcomes. Therefore, the aim of this study was to determine the long-term health outcome (≥10 years) of QFS, both for the total QFS patient population and separately for three different age groups of patients: <40 years, ≥40–< 65 years, and ≥ 65 years.

## Materials and methods

### Study design and participants

This study was performed in collaboration with Q-support, the Q-fever expertise, and a support centre for patients and healthcare professionals in the Netherlands [12]. This study describes the baseline measurement of a large longitudinal study aiming at improving insights into the long-term impact of QFS on patient’s health, well-being, social participation, and healthcare use. In September 2021, adult QFS patients registered at Q-support received an email invitation to fill out an online questionnaire about the impact of QFS on their lives. The questionnaire was developed in collaboration with healthcare providers and QFS patients (Supplementary material S1). Patients could request a paper version of the questionnaire. If patients did not respond within two weeks, an email reminder was sent. A second reminder was sent if patients did not respond within two weeks of the first reminder; a third reminder was sent if patients did not respond within two weeks of the second reminder.

Study participation was voluntary, and all participants provided online informed consent before filling out the questionnaire. The study was approved by the Medical Ethics Review Board of Erasmus MC (MEC-2021-1606), conducted in line with the principles of the Declaration of Helsinki, and followed the STROBE guidelines. For this manuscript, patients ≥10 years post-Q-fever infection were selected. In case where the year of the Q-fever infection was unknown, the year of the QFS diagnoses was used as a substitute. In case where the year of QFS diagnoses was also unknown, patients were excluded.

### Measures

#### Socio-demographic and medical characteristics

Questions on socio-demographic and medical characteristics were included in the questionnaire. Socio-demographic characteristics included age, gender, educational level, and living situation. Age was categorised into young (<40 years), middle-aged (≥40–< 65 years), and older (≥65 years). These age subgroups conform to the age distribution used by Statistics Netherlands [13]. Educational level was categorised into low, middle, and high according to the International Standard Classification of Education [14]. The living situation was dichotomised as living alone and not living alone for regression analyses.

Medical characteristics were self-reported and included year of Q-fever infection, year of QFS diagnosis, use of antibiotics and hospitalisation during the acute infection, and the number of hospitalisations since infection. Furthermore, patients indicated whether they had co-existing chronic diseases from a list of fourteen chronic diseases, or supplemented the list with any other chronic via an open-answer option.

#### Long-term health complaints

The questionnaire included a list of 30 health complaints related to Q-fever and/or QFS, which was composed based on the available literature and experts in the field. Patients were asked to indicate which of these 30 complaints they had experienced since the acute Q-fever infection.

#### Health-related quality of life

Generic HRQL on the day of filling out the questionnaire was measured with the EQ-5D-5L + cognition (EQ-5D-5L + C) [15]. This instrument consists of the original five EQ-5D dimensions, mobility, self-care, usual activities, pain/discomfort, and anxiety/depression, and includes an extra cognition dimension. For each dimension, patients indicated whether they experienced no problems, slight problems, moderate problems, severe problems, or extreme problems on the day of completing the questionnaire. Based on the five original dimensions and using the Dutch value set, the EQ-5D index was calculated, ranging from 0 (for a health state considered as bad as being dead) to 1 (full health) [16]. In addition, participants scored their overall health status on a visual analogue scale (EQ-VAS), which ranges from 0 (worst imaginable health) to 100 (best imaginable health).

#### Energy, fatigue, and post-exertional malaise

Patients were asked to estimate their current energy level compared with before their Q-fever infection. Fatigue in the previous days was measured with the Multidimensional Fatigue Inventory (MFI) [17]. This instrument includes 20 items on five different scales: general fatigue, physical fatigue, reduction in activity, reduction in motivation, and mental fatigue. Items are scored on a 5-point Likert scale ranging from 1 (‘yes, that is true’) to 5 (‘no, that is not true’). Item scores are summed into scale scores ranging from 4 to 20; higher scores indicate more fatigue [17]. The MFI-20 total score was calculated as the sum of all MFI items, ranging from 20 to 100.

Post-exertional malaise (PEM) in the previous days was assessed using the five items for post-exertional neuroimmune exhaustion [18]. Patients scored items on a 5-point Likert scale ranging from 1 (‘yes, that is true’) to 5 (‘no, that is not true’).

#### Mental health

Depression in the previous two weeks was measured with the two-item version of the Patient Health Questionnaire-9 (PHQ-2) [19]. Anxiety in the previous two weeks was measured with the two-item version of the Generalized Anxiety Disorder-7-NL-4 (GAD-2) [20]. Items of the PHQ-2 and GAD-2 were scored on a 4-point Likert scale, ranging from 0 (‘do not suffer from these problems at all’) to 3 (‘suffer almost every day’). Total scores for the PHQ-2 and GAD-2 ranged from 0 to 6. A score of ≥3 indicates that anxiety or depression is likely [19, 20]. Depression (PHQ-2) and anxiety (GAD-2) outcomes were dichotomised based on the cut-off point.

### Statistical analysis

Descriptive statistics were used to describe the socio-demographic and medical characteristics and outcomes of the total study sample and age subgroups [13]. Continuous data were reported as mean (SD) if normally distributed and as median (IQR) if not normally distributed. Categorical data were reported as numbers (percentage).

Depending on the normality of the distribution, ANOVA or Kruskal–Wallis H tests were used to test differences in scores of continuous variables between the three age subgroups. The chi-square test was used to compare the distribution of categorical variables, except for small numbers (n < 5), and then, Fisher’s exact test was used. If appropriate, when the test result was significant for the comparison among the three age subgroups, Bonferroni’s tests were used for multiple comparisons.

To study determinants associated with the number of long-term health complaints, HRQL, and health status, fatigue univariate linear regression was applied. EQ-5D index score (HRQL) and total MFI-20 score (fatigue) outcomes were transformed to a 0–100 scale to make outcomes comparable and easier to interpret: 0 represents the worst outcome and 100 represents the best outcome. Determinants associated with PEM, anxiety, and depression were studied using logistic regression. For PEM, item 1 (‘Marked, rapid physical and/or cognitive fatigability in response to exertion’) was used, which was dichotomised into ‘no PEM’ (score 4–5) and ‘any PEM problems’ (score 1–3). Socio-demographic and medical characteristics were included as independent variables. The collinearity (>0.8 or < −0.8) of all determinants with a p-value <0.10 in univariate analyses was checked. The remaining determinants were used in multivariate analyses. Regression coefficients and standard errors were presented. A *P*-value of <0.05 was considered statistically significant. We used IBM SPSS Statistics 28 for all analyses.

## Results

### Patient characteristics

A total of 842 patients were invited to participate, of whom 457 completed the questionnaire (response rate: 54.3%) (Supplementary material S2). Eighty-nine patients were excluded because they did not meet the inclusion criteria, resulting in the inclusion of 368 patients. The main reason for exclusion was a Q-fever infection less than 10 years ago.

The median age of patients was 57 years (IQR: 49.0–64.0), and 54.1% were female (Table 1). The majority of patients lived together (74.4%), and many of them had comorbidity (66.3%). The median time since Q-fever infection was 12 years (IQR: 12.0–13.0). During the acute phase of the Q-fever infection, 63.6% of the patients received antibiotics and 16.0% were hospitalised. The three age subgroups differed statistically significantly on gender, level of education, household composition, comorbidity, and use of antibiotics during the acute phase of the infection (Table 1). In particular, the youngest age group had a larger proportion of females (79.1%), and the lowest proportion of patients had a co-existing chronic disease (37.2%).

**Table 1.** Socio-demographic and medical characteristics of chronically fatigued and developed Q-fever fatigue syndrome assisted at Q-support Netherlands, 2021

		Total sample (*n* = 368)	Patients < 40 years old (*n* = 43)	Patients 40–<65 years old (*n* = 237)	Patients ≥ 65 years old (*n* = 88)	*P*-value for difference
Age at the time of study, median (IQR)		57.0 (49.0–64.0)	32.0 (29.0–35.0)	54.0 (49.5–60.0)	69.0 (66.0–72.0)	
Age at the time of infection, median (IQR)		44.0 (36.0–51.0)	20.0 (17.0–23.0)	42.0 (37.0–48.0)	57.0 (54.0–60.0)	
Gender						<0.001
Male		169 (45.9%)	9 (20.9%)	110 (46.4%)	50 (56.8%)	
Female		199 (54.1%)	34 (79.1%)	127 (53.6%)	38 (43.2%)	
Level of education						<0.001
Low		102 (27.7%)	3 (7.0%)	69 (29.1%)	30 (34.1%)	
Middle		149 (40.5%)	22 (51.2%)	106 (44.7%)	21 (23.9%)	
High		117 (31.8%)	18 (41.9%)	62 (26.2%)	37 (42.0%)	
Household composition						<0.001
Married or living together without children living at home		159 (43.2%)	7 (16.3%)	76 (32.1%)	76 (86.4%)	
Married or living together with children living at home		111 (30.2%)	19 (44.2%)	88 (37.1%)	4 (4.5%)	
One-parent household without children living at home		6 (1.6%)	0 (0.0%)	5 (2.1%)	1 (1.1%)	
One-parent household with children living at home		14 (3.8%)	1 (2.3%)	12 (5.1%)	1 (1.1%)	
Living alone		61 (16.6%)	10 (23.3%)	46 (19.4%)	5 (5.7%)	
Other		17 (4.6%)	6 (14.0%)	10 (4.2%)	1 (1.1%)	
Comorbidity						<0.001
No co-existing chronic disease		124 (33.7%)	27 (62.8%)	77 (32.5%)	20 (22.7%)	
≥1 co-existing chronic disease		244 (66.3%)	16 (37.2%)	160 (67.5%)	68 (77.3%)	
Years since Q-fever infection, median (IQR)		12.0 (12.0–13.0)	12.0 (11.0–12.0)	12.0 (12.0–13.0)	12.0 (12.0–13.0)	0.883
Before 2007		19 (5.2%)	1 (2.3%)	14 (5.9%)	4 (4.5%)	
Between 2007 and 2011		349 (94.8%)	42 (97.7%)	223 (94.1%)	84 (95.5%)	
Antibiotics during the acute phase of the infection						0.04
Yes		234 (63.6%)	19 (44.2%)	158 (66.7%)	57 (64.8%)	
No		111 (30.2%)	18 (41.9%)	66 (27.8%)	27 (30.7%)	
Not sure		23 (6.3%)	6 (14.0%)	13 (5.5%)	4 (4.5%)	
Hospitalisation during the acute phase of the infection						0.127
Yes		59 (16.0%)	3 (7.0%)	44 (18.6%)	12 (13.6%)	
No		309 (84.0%)	40 (93.0%)	193 (81.4%)	76 (86.4%)	

### Long-term health complaints

**Figure 1.**
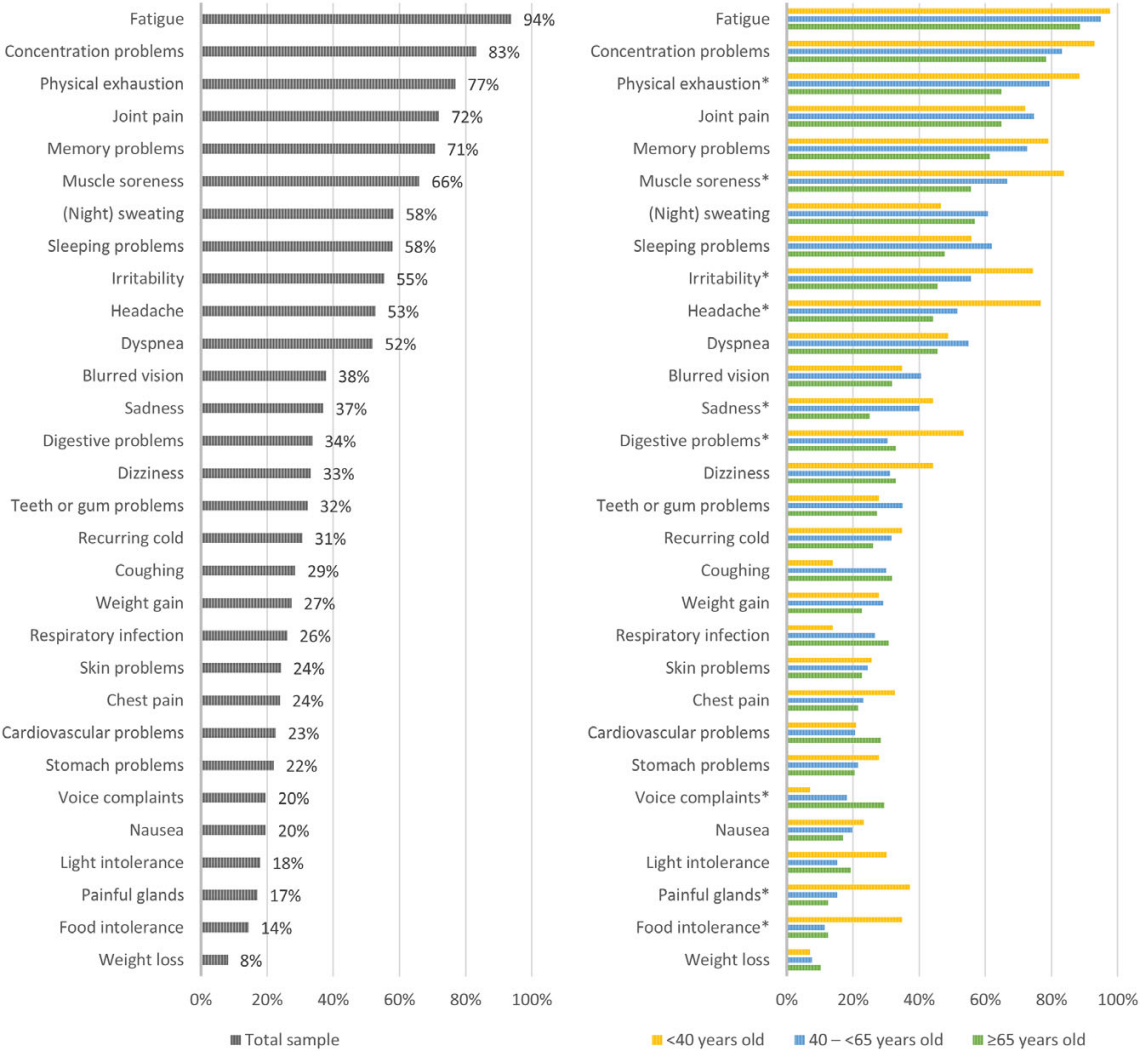
Percentage of Q-fever fatigue syndrome patients that reported a specific health complaint, for the total sample (a) and for subgroups of patients based on their age (b). *Indicating statistically significant differences (*P* < 0.05) among the three age subgroups.

For the total study sample, the median number of long-term health complaints was 12.0 (IQR: 8.0–16.0) out of a predefined list of thirty complaints (Table 2). All patients reported one or more health complaints, with a few patients (10.1%) reporting ≤5 complaints. The most reported health complaint was fatigue (93.8%), followed by concentration problems (83.2%) and physical exhaustion (76.9%) (Figure 1a). Many patients (64.1%) reported all three complaints.

**Table 2.** Long-term health outcomes for Q-fever fatigue syndrome patients assisted at Q-support Netherlands, 2021

	Total (*n* = 368)	Patients < 40 years old (*n* = 43)	Patients 40–<65 years old (*n* = 237)	Patients ≥ 65 years old (*n* = 88)	*P*-value for difference
	Median (IQR)	Median (IQR)	Median (IQR)	Median (IQR)	
Number of long-term complaints	12.0 (8.0–16.0)	13.0 (9.0–18.0)	12.0 (8.0–16.0)	11.0 (7.0–14.0)	0.043
Health-related quality of life					
EQ-5D-5L index	0.63 (0.38–0.75)	0.67 (0.41–0.74)	0.61 (0.33–0.74)	0.68 (0.50–0.78)	0.018
EQ-VAS	50.0 (34.3–61.8)	40.0 (30.0–60.0)	46.0 (30.0–60.0)	60.0 (41.5–70.0)	<0.001
Fatigue					
MFI-20 total score	74.0 (65.0–84.0)	77.0 (66.0–83.0)	76.0 (67.0–85.0)	69.0 (61.3–76.0)	<0.001
General fatigue	18.0 (15.0–20.0)	19.0 (18.0–20.0)	18.0 (15.0–20.0)	16.0 (14.0–19.0)	<0.001
Physical fatigue	16.0 (14.0–19.0)	17.0 (15.0–19.0)	16.0 (14.0–19.0)	15.0 (13.0–17.0)	0.001
Reduction in activity	14.0 (12.0–17.0)	14.0 (10.0–17.0)	15.0 (12.0–17.0)	14.0 (10.0–16.0)	0.029
Reduction in motivation	12.0 (9.3–15.0)	11.0 (9.0–14.0)	12.0 (10.0–15.0)	12.0 (8.3–13.0)	0.013
Cognitive fatigue	14.0 (12.0–17.0)	15.0 (12.0–18.0)	15.0 (12.0–17.5)	13.0 (11.0–16.0)	<0.001
Depression					0.018
PHQ-2 score	2.0 (1.0–3.0)	2.0 (1.0–2.0)	2.0 (1.0–3.0)	1.0 (0.0–2.0)	
Depression likely (score ≥ 3) (*n* (%))	101 (27.4%)	8 (18.6%)	80 (33.8%)	13 (14.8%)	
Anxiety					0.025
GAD-2 score	1.0 (0.0–2.0)	1.0 (0.0–3.0)	1.0 (0.0–2.0)	0.0 (0.0–2.0)	
Anxiety likely (score ≥ 3) (*n* (%))	79 (21.5%)	11 (25.6%)	57 (24.1%)	11 (12.5%)

Younger patients (<40 years) reported a median of 13.0 (IQR: 9.0–18.0) health complaints; middle-aged patients (40 – <65 years) reported a median of 12.0 (IQR: 8.0–16.0) health complaints; and older patients (≥65 years) reported a median of 11.0 (IQR: 7.0–14.0) health complaints. The top three complaints were identical for all three age subgroups studied. The prevalence of nine of thirty complaints was statistically significantly different among the groups (Figure 1b). The prevalence of headache, painful glands, and food intolerance was higher in younger patients than in middle-aged and older patients (*P* < 0.001 – *P* = 0.006). Muscle soreness and irritability were more often reported by young compared with old patients (*P* = 0.004–0.005) and digestive problems by young versus middle-aged patients (*P* = 0.009). Both young and middle-aged patients experienced more physical exhaustion than older patients (*P* = 0.008–0.016), and sadness was more often reported by middle-aged patients compared with older patients (*P* = 0.037). Voice complaints were more prevalent among older patients compared with young patients (*P* = 0.007).

### Health-related quality of life

**Figure 2.**
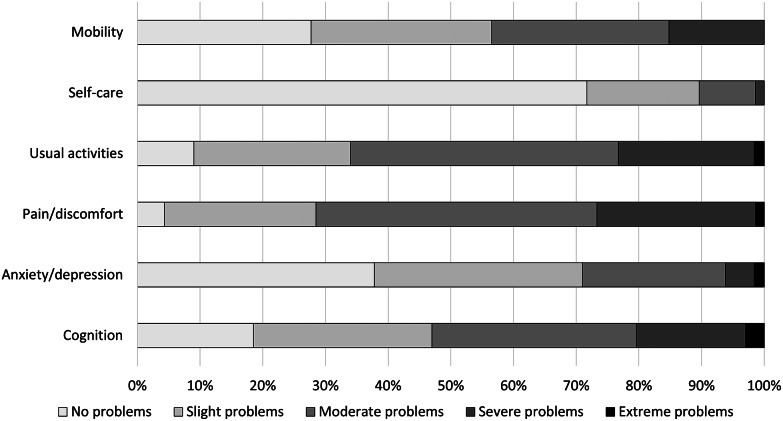
Frequency of responses to the EQ-5D-5L + C dimensions for the total sample of patients.

**Figure 3.**
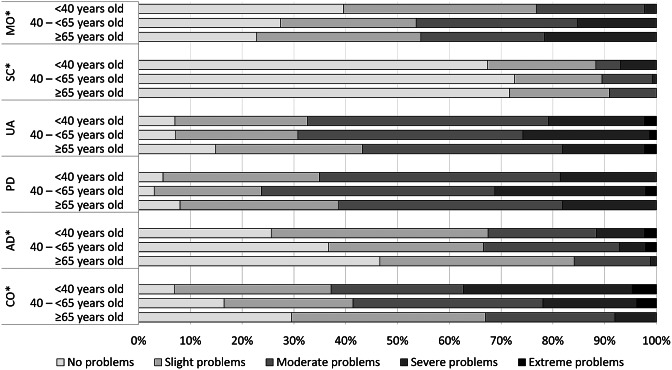
Frequency of responses to the EQ-5D-5L + C dimensions for subgroups of patients based on age. AD, anxiety/depression; CO, cognition; MO, mobility; PD, pain/discomfort; SC, self-care; UA, usual activities. *Indicating statistically significant differences (*P* < 0.05) among the three age subgroups.

The median EQ-5D-5L index for the total study sample was 0.63 (IQR: 0.38–0.75; mean: 0.56 (SD: 0.25)), and the median EQ-VAS was 50.0 (IQR: 34.3–61.8; mean: 49.3 (SD: 19.4)) (Table 2). Many patients reported any problems on the dimensions of pain/discomfort (95.7%) and usual activities (91.0%) (Figure 2). HRQL (EQ-5D-5L index score) was lowest for the middle-aged group (median: 0.61; IQR: 0.33–0.74) and statistically significantly worse compared with the oldest age group (median: 0.67; IQR: 0.50–0.79; *P* = 0.018). Health status (EQ-VAS score) was lowest for the youngest age group (median: 40.0; IQR 30.0–60.0) and highest for individuals ≥65 years old (median: 60.0; IQR: 41.5–70.0). The health status was statistically significantly better for the oldest age group compared with both the young and middle-aged subgroups (*P* < 0.001). All dimensions, except for pain/discomfort and usual activities, were scored significantly different among the age subgroups (Figure 3). In particular, the proportion of patients experiencing problems with cognition was considerably higher for younger patients (93.0%) versus middle-aged (83.5%) and old patients (70.5%) (*P* < 0.001).

### Energy, fatigue, and post-exertional malaise

Compared to before the Q-fever infection, as reported via recall in the questionnaire, patients had a median energy level of 45.0% (IQR: 30.0–60.0%). This percentage was not statistically significantly different among the subgroups (*P* = 0.179). The median level of fatigue (MFI-20 score) was high (median: 77.0; IQR: 65.0–84.0), with patients scoring worst on the domain ‘general fatigue’ (median: 18.0; IQR: 15.0–20.0) and relatively best on the domain ‘reduction in motivation’ (median: 12.0; IQR: 9.3–15.0) (Table 2). Older patients had a statistically significantly lower MFI-20 fatigue score and lower scores for all domains than young and middle-aged patients (*P* < 0.001), indicating relatively less fatigue problems in the oldest age group (Table 2).

**Figure 4.**
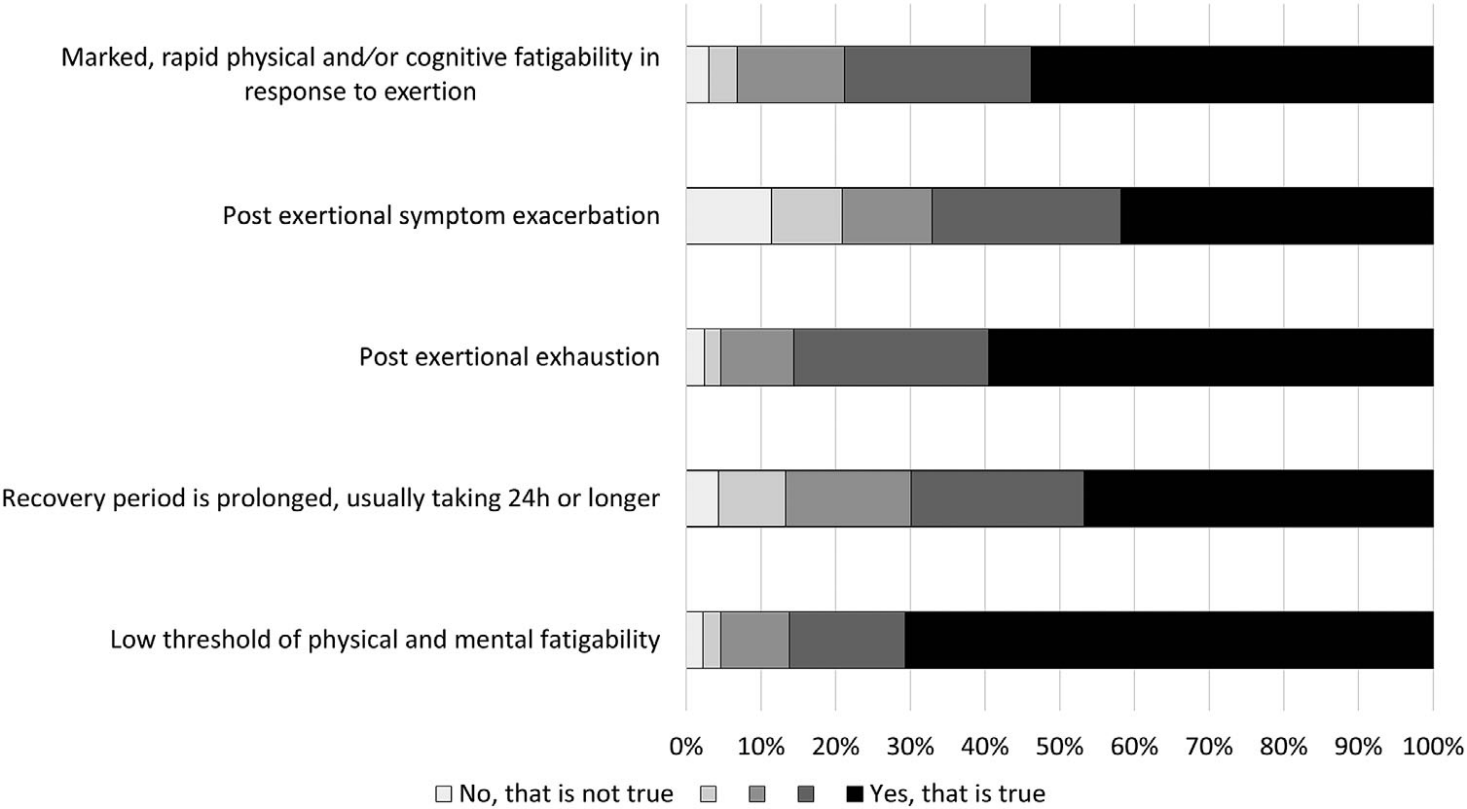
Frequency of responses to the post-exertional malaise (PEM) dimensions for the total sample of patients.

**Figure 5.**
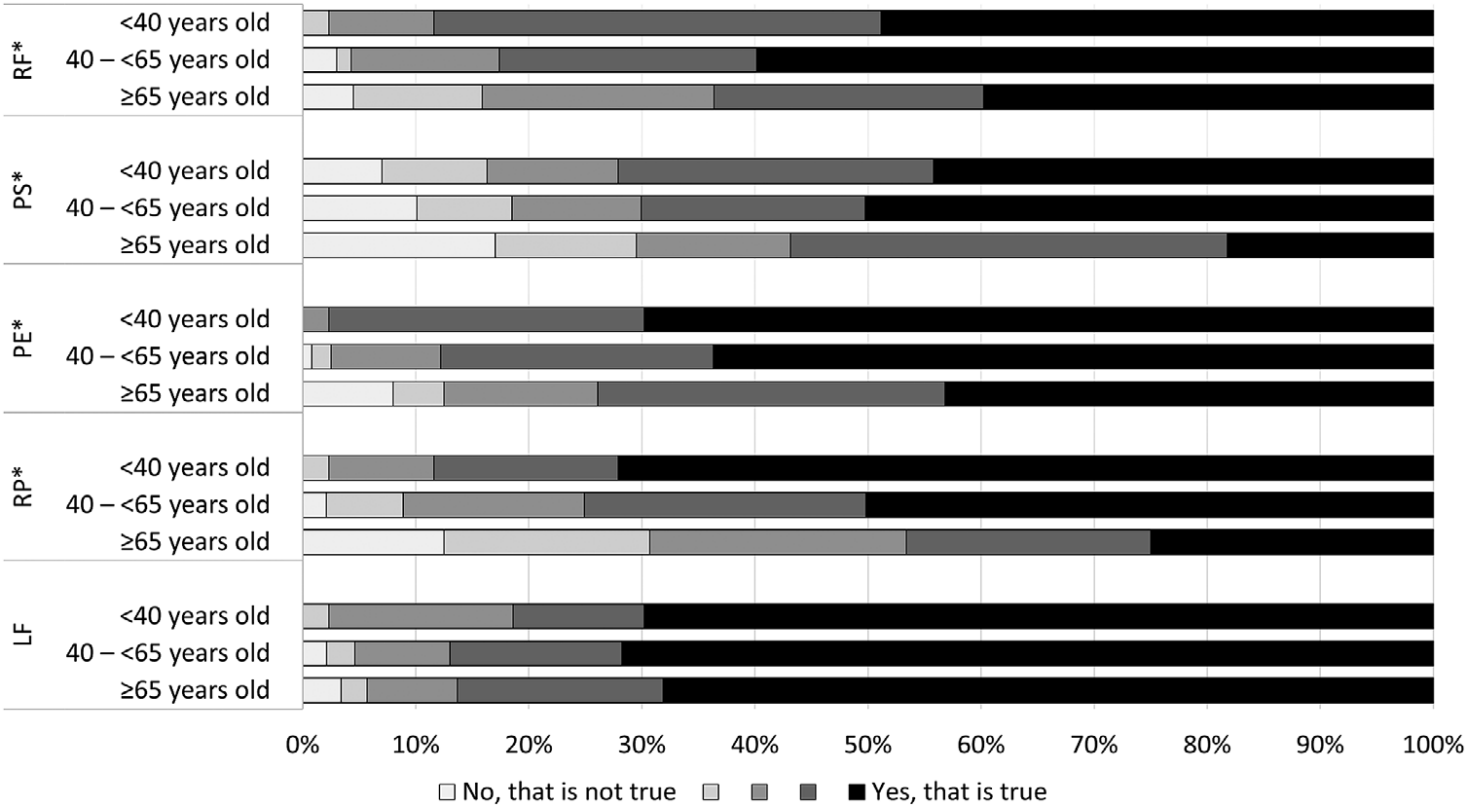
Frequency of responses to the post-exertional malaise (PEM) dimensions for subgroups of patients based on age. *Indicating statistically significant differences (*P* < 0.05) among the three age subgroups. LF, low threshold of physical and mental fatigability; PE, post-exertional exhaustion; PS, post-exertional symptom exacerbation; RF, marked, rapid physical, and/or cognitive fatigability in response to exertion; RP, recovery period is prolonged, usually taking 24 h or longer.

Post-exertional malaise complaints were common in QFS patients; all (98.9%) except four patients reported at least mild complaints in one of the five post-exertional malaise domains. Almost all patients reported at least mild complaints (95.6–97.8%) on four of the post-exertional malaise domains, and many also reported at least mild post-exertional symptom exacerbation (88.6%) (Figure 4). Older patients reported statistically significantly less post-exertional malaise complaints (Figure 5; *P* < 0.001–*P* = 0.032), except for the domain ‘low threshold of physical and mental fatigability’, for which patients in all subgroups reported many problems (96.7–100%). No significant differences were found between the younger and middle-aged patient groups.

### Mental health

The median depression score for all patients was 2.0 (IQR: 1.0–3.0) (Table 2). In total, 27.4% of all QFS patients had a score that is indicative of having depression. Over half of those patients (n = 56; 55.4%) also had a score that is indicative of having anxiety. The proportion of patients with likely depression was statistically significantly higher in middle-aged patients (33.8%) than in the oldest patients (14.8%; *P* < 0.001); no statistically significant differences were found for the other age group comparisons (Table 2).

QFS patients had a median anxiety score of 1.0 (IQR: 0.0–2.0), and 21.5% of the patients had a score that is indicative of having anxiety (Table 2). The young age group had the highest proportion of patients likely having anxiety (25.6%). The proportion of patients likely having anxiety was statistically significantly higher in the middle-aged group (24.1%) than in the oldest age group (12.5%; *P* = 0.006).

### Determinants of long-term health outcomes

Univariate and multivariate predictive determinants of long-term health outcomes are presented in Supplementary material S3 and Supplementary Table S3. Older age (≥65 years) was universally significantly associated with better outcomes in univariate analyses. In multivariate analyses, older age remained associated with better outcomes for all health outcomes studied, except for the number of long-term health complaints (Tables 3 and 4). Other determinants in the univariate analyses were less universal and differed per outcome studied. In multivariate analyses, living alone was associated with an increased number of long-term health complaints; not having a co-existing chronic disease with a better HRQL and a better health status; males had a higher risk of depression; and having a low education was associated with an increased risk of having anxiety.

**Table 3.** Multivariate linear regression analyses for long-term health complaints, health-related quality of life, and fatigue

		Health complaints		Health-related quality of life				Fatigue	
		Number of long-term complaints		Transformed EQ-5D-5L index		EQ-VAS		Transformed MFI-20 score	
		Coef.	*P*-value	Coef.	*P*-value	Coef.	*P*-value	Coef.	*P*-value
Age at the time of study									
<40 years old									
40–<65 years old (reference)									
≥65 years old				9.645	**0.001**	11.798	**<0.001**	7.305	**<0.001**
Gender									
Male									
Female (reference)									
Education									
Low									
Middle (reference)									
High									
Living situation									
Not living alone (reference)									
Living alone		1.868	**0.009**						
Comorbidity									
No co-existing chronic disease				10.578	**<0.001**	5.674	**0.007**		
≥1 co-existing chronic disease (reference)									
Antibiotics									
Yes (reference)									
No									
Not sure									
Hospitalisation during the acute phase of infection									
Yes									
No (reference)									

Note. *P*-values printed in bold indicate statistically significant values (*P* < 0.05).

Abbreviations: EQ-VAS, EQ visual analogue scale; MFI-20, multidimensional fatigue inventory 20-item version.

**Table 4.** Multivariate logistic regression analyses for post-exertional malaise, depression, and anxiety

		Post-exertional malaise		Depression		Anxiety	
		Item 1		PHQ-2		GAD-2	
		OR	*P*-value	OR	*P*-value	OR	*P*-value
Age at the time of study							
<40 years old							
40–<65 years old (reference)							
≥65 years old		0.216	**<0.001**	0.323	**<0.001**	0.409	**0.012**
Gender							
Male				2.412	**<0.001**		
Female (reference)							
Education							
Low						2.137	**0.005**
Middle (reference)							
High							
Living situation							
Not living alone (reference)							
Living alone							
Comorbidity							
No co-existing chronic disease							
≥1 co-existing chronic disease (reference)							
Antibiotics							
Yes (reference)							
No							
Not sure							
Hospitalisation during the acute phase of infection							
Yes							
No (reference)							

Note. *P*-values printed in bold indicate statistically significant values (*P* < 0.05).

Abbreviations: GAD-2, generalized anxiety disorder 2-item version; PEM item 1, marked, rapid physical, and/or cognitive fatigability in response to exertion; PHQ-2, patient health questionnaire 2-item version.

## Discussion

The present study determined the long-term health outcomes of patients living with QFS and the determinants associated with health outcomes. In general, QFS patients experience a wide range of health complaints, high levels of fatigue and PEM, a low HRQL, and an increased risk of depression and anxiety. Almost all patients reported experiencing at least slight pain, and the usual activities of nine of ten patients were limited to some extent. Patients’ median level of energy was less than half compared with before the Q-fever infection. Generally, young and middle-aged patients seemed to experience more long-term consequences compared with older patients, and they reported significantly worse health status, higher fatigue levels and anxiety, and more post-exertional malaise complaints; middle-aged patients have a lower HRQL and a higher risk of depression. Multivariate regression analyses confirmed that older age was associated with better health outcomes, except for the number of long-term health complaints.

The HRQL of QFS patients in our sample was low, which is in line with earlier studies that reported a diminished HRQL of QFS patients up to 10 years post-infection [8, 10]. The HRQL of QFS patients was considerably lower than the HRQL of the Dutch population (mean EQ-5D index: 0.56 versus 0.89), indicating the large impact of QFS on patients’ HRQL [21]. Patients’ health status was also severely impacted and much lower compared with the norm score for the Netherlands (mean EQ-VAS: 49.3 vs 82.0) [21]. The largest discrepancies in reported problems between the QFS patients and the Dutch general population were on the dimensions of usual activities and pain/discomfort [21]. However, these norm scores are only available for the EQ-5D-3L, which impacts comparability as research shows that the 3 L version of the instrument is less sensitive, especially for mild problems [22]. When comparing results to a large study in the general Dutch population with the EQ-5D-5L (mean EQ index: 0.83), the QFS patients still have a substantially worse HRQL, also in comparison with patients with a chronic health condition (mean EQ index: 0.73) corroborating the large impact of QFS on patients’ HRQL [23, 24].

The high number and large diversity of health complaints, and the more than fifty per cent energy reduction compared with pre-infection may explain the diminished HRQL. Patients experienced, on average, twelve different health complaints, and the proportion of patients experiencing fatigue was considerably higher than in the Dutch general population [23]. In line with earlier studies, it was found that fatigue was the most frequent and most severe health complaint for QFS patients [6, 8, 9, 25]. Being fatigued has been shown to be associated with lower HRQL in other patient populations [26]. Concentration problems and physical exhaustion were also among the three most prevalent health complaints. Strikingly, more than half of the patients (64.1%) experienced all three complaints. An earlier study reported the identical top three most prevalent complaints up to 10 years post-infection [8]. These long-lasting complaints thus seem to remain and can be considered chronic health complaints. The median number of total health complaints was somewhat higher in the previous study (median 13 of 27 versus 12 of 30 complaints in our study), possibly indicating that the number of total complaints slightly decreased over time. However, this should be confirmed in a longitudinal study. It might also be caused by the different study sample or the slightly different list of complaints.

In addition to these health complaints, the majority of patients experienced severe PEM problems. This is in line with recent studies that showed that PEM is an important and severe health complaint in QFS patients and in other patient populations that experience long-term sequelae of infectious diseases [27]. Also, patients who provided input for our study indicated that PEM is a relevant but underexposed theme for them. The impact and importance of PEM for QFS patients were underlined by our study results.

Mental health problems were less prevalent. Over a quarter of QFS patients had scores indicative of depression, which is more than twice the prevalence in the Dutch general population (27.4% vs 12.6%). Also, the percentage of patients likely having anxiety was almost two times higher (21.5% vs 11.0%) [28]. It is somewhat questionable whether these anxiety and depression rates were fully representable as they were measured during the COVID-19 pandemic; COVID-19 has a proven negative effect on anxiety and depression rates [29, 30]. However, the comparison rates used were also assessed during the COVID-19 pandemic, though one year earlier (April–May 2020) [28]. Similar to other patient populations, depression and anxiety frequently co-occurred in QFS patients; over half of the patients in our study who had a score indicative of depression also had a score indicative of anxiety [31].

Our results suggest large differences in health outcomes between subgroups of patients based on age. In general, young and middle-aged QFS patients seem to have worse health outcomes than older patients. However, all patients, including the older patients, have substantially worse HRQL, health status, level of fatigue, and increased risk of anxiety and depression compared with their counterparts without QFS [21, 28]. Our results might be somewhat surprising, as, generally, older age is associated with more comorbidity, higher levels of fatigue, and a lower HRQL [21]. However, our results might be explained by the fact that people have different social roles in different stages of life. Young and middle-aged patients have more clearly defined social roles, such as education, work and/or parenting, and in particular, younger patients (aged <40 years) have to perform an extensive range of social roles. Younger and middle-aged patients might experience more problems as they have, in general, a busier life and less time to recover than older, retired people with less specified roles, more time to rest, and a slower pace of life. Multivariate regression analyses confirmed that older age was associated with better health outcomes, except for the number of long-term health complaints. However, these findings should be interpreted with some caution as the number of patients was not evenly divided among the three subgroups studied. Patients 40–65 years old represented a large part of our study sample, as they do in the total Q-support QFS population. Also, differences in characteristics in the three age subgroups might have influenced the differences in outcomes. The youngest age group had a larger proportion of females, many patients with a high level of education, and the lowest proportion of patients with a co-existing chronic disease. The literature shows that, in general, females tend to experience more health complaints, or are more willing or open to report health complaints, which may have led to an overrepresentation of long-term complaints [32]. In contrast, earlier studies indicate that low education is associated with impaired health outcomes and HRQL [33, 34]; this might have led to the underrepresentation of long-term complaints in the youngest age group. The oldest age group had, as expected, the highest proportion of patients with comorbidity, which is generally associated with more long-term complaints [21].

In our study, not having a co-existing chronic disease was associated with a better HRQL and health status, which is in line with the existing literature that describes a strong relationship between multimorbidity and diminished HRQL [35]. Hospitalisation and antibiotic treatment during the acute phase of the Q-fever infection were not associated with less severe long-term health outcomes in QFS patients in our study. This is in line with previous research that concluded that long-term doxycycline use did not reduce the severity of health outcomes [36]. Furthermore, our results showed an association between being male and a higher likelihood of depression. This is in contrast with the findings of large meta-analyses on depression in representative national samples from over 90 countries that reported that there are roughly twice as many females with depression as males with depression [37]. The relation found in our study might be provoked by the fact that many QFS patients are severely limited in their daily activities, including their work activities, which might have a stronger impact on males’ mental health as males are traditionally considered the main wage earner in a household.

This study had several strengths, including a large cohort of QFS patients and information on various determinants and health outcomes, enabling us to study a variety of health outcomes in specific subgroups of patients. Also, the long-term aspect of our study was unique, and considering the impaired health and functioning of patients, which may have hampered participation in this study, the response rate (54.3%) was high. However, some limitations were also present, like the enrolment of solely patients registered at Q-support in this study. Even though Q-support is the Q-fever expertise and support centre for patients in the Netherlands, the patients registered at Q-support may not be representative of all QFS patients in the Netherlands. Besides, we were unable to perform a non-response analysis as information on time since infection was not available. We were therefore not able to study whether responder’s characteristics differed from those of non-responders. Due to the long follow-up period, there is a risk of recall bias in our results regarding the situation before and during the acute Q-fever infection [38]. Another limitation is the cross-sectional study design that hampered the insight into the course of health complaints and health outcomes over time and determinants for worse health outcomes. Furthermore, it is possible that patients with severe health problems have not completed the full questionnaire, which may have led to underestimating the severity of the problems. However, this may have been countered partly by QFS patients without or with few complaints who did not participate in the questionnaire, for example because they have less time or less interest to complete a questionnaire on a disease that is barely impacting their lives. Also, the uneven distribution of patients in the age categories might be considered a limitation, as well as the statistically significant differences in characteristics among the three age subgroups, which might have impacted our results. Besides, in our regression analyses for the EQ-5D index, not all assumptions for linear regression were met; therefore, these results have been presented with somewhat more caution but still provide a good insight into the relationship with associated determinants. Lastly, the questionnaires used for fatigue and PEM might have been suboptimal for our population, as a recently published study showed that the MFI-20 has a questionable factor structure in the general Dutch population [39], and the PEM questions are based on diagnostic criteria [18], whereas a brief questionnaire for PEM has been recently developed and might be a better option to use for future studies [40].

## Conclusions

QFS has a considerable impact on patients’ health for more than 10 years after the Q-fever infection. Patients have to live and cope with a wide range of health complaints, high levels of fatigue, and diminished HRQL. This emphasises not only the long-term negative impact on patients but also the complexity of the treatment of health complaints by healthcare providers. Young and middle-aged QFS patients experience more long-term consequences compared with older patients. Besides, males tend to have an increased risk of depression. Tailored health care is recommended to provide optimal care and support for each QFS patient, keeping these age-specific health consequences in mind.

## Supporting information

Spronk et al. supplementary material Spronk et al. supplementary material

## Data Availability

The data set used and analysed during this study is available from the corresponding author on reasonable request.

## List of abbreviations

HRQL: health-related quality of life
PEM: post-exertional malaise
QFS: Q-fever fatigue syndrome

## Supplementary material

For supplementary material accompanying this paper visit http://doi.org/10.1017/S0950268823001401. click here to view supplementary material
