# Supplementary material for: Long-term health outcomes of Q-fever fatigue syndrome patients
Source: Epidemiol Infect. 2023 Sep 19;151:e179. doi: 10.1017/S0950268823001401 (PMC10600735; doi:10.1017/S0950268823001401)
Supplement: Spronk et al. supplementary material [file S0950268823001401sup001.docx]

**APPENDIX A: Table A1. Variables in questionnaire**

**Patient characteristics:**

- Age in years
- Gender
- Level of education
- Household composition
- Type of comorbidity
- Year of Q-fever infection
- Year of QFS diagnosis
- Hospital admission during acute phase of Q-fever infection
- Antibiotics during acute phase of Q-fever infection

**Symptoms since Q-fever infection:**

- Blurred vision
- Cardiovascular problems
- Chest pain
- Concentration problems
- Coughing
- Digestive problems
- Dizziness
- Dyspnea
- Fatigue
- Food intolerance
- Headache
- Irritability
- Joint pain
- Light intolerance
- Memory problems
- Muscle soreness
- Nausea
- (Night) sweating
- Painful glands
- Physical exhaustion
- Recurring cold
- Respiratory infection
- Sadness
- Skin problems
- Sleeping problems
- Stomach problems
- Teeth or gum problems
- Voice complaints
- Weight gain
- Weight loss

**Other health outcomes:**

- EQ-5D-5L (5 items, extra item cognition, and visual analogue scale)
- Energy level compared to before Q-fever infection
- Multidimensional Fatigue Inventory
- Five items on post exertional neuroimmune exhaustion
- Patient Health Questionnaire 2-item
- Generalized Anxiety Disorder 2-item

**APPENDIX B**

**
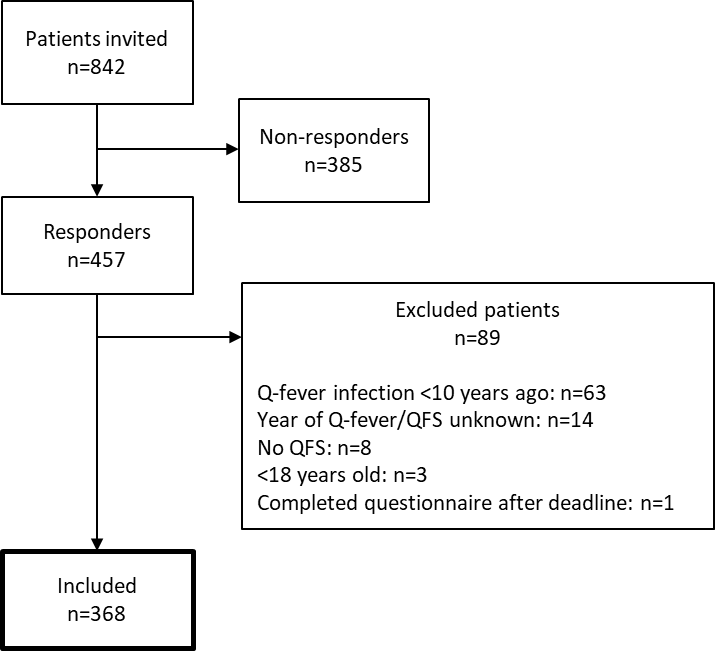
**

**Figure B1.** Flowchart of patient selection.

**APPENDIX C**

**Table C1.** Univariate linear regression analyses of long-term health complaints, health-related quality of life and fatigue.

|  | | Health complaints | | Health-related quality of life | | | | Fatigue | |
| --- | --- | --- | --- | --- | --- | --- | --- | --- | --- |
|  | | Number of long-term complaints | | Transformed EQ-5D-5L index | | EQ VAS | | Transformed MFI-20 score | |
|  | | Coef. | p-value | Coef. | p-value | Coef. | p-value | Coef. | p-value |
| Age at time of study | |  |  |  |  |  |  |  |  |
|  | <40 years old | 1.624 | 0.069 | 1.829 | 0.654 | -5.309 | 0.091 | -1.537 | 0.487 |
|  | 40 - <65 years old (reference) |  |  |  |  |  |  |  |  |
|  | ≥65 years old | -1.388 | **0.039** | 8.120 | **0.008** | 10.980 | **<0.001** | 7.305 | **<0.001** |
| Gender | |  |  |  |  |  |  |  |  |
|  | Male | -0.928 | 0.107 | -0.782 | 0.766 | 0.355 | 0.861 | -1.112 | 0.435 |
|  | Female (reference) |  |  |  |  |  |  |  |  |
| Education | |  |  |  |  |  |  |  |  |
|  | Low | -1.139 | 0.076 | -1.129 | 0.700 | -1.491 | 0.509 | -2.579 | 0.104 |
|  | Middle (reference) |  |  |  |  |  |  |  |  |
|  | High | -0.127 | 0.837 | 3.824 | 0.174 | 4.269 | **0.049** | 2.707 | 0.075 |
| Living situation | |  |  |  |  |  |  |  |  |
|  | Not living alone (reference) |  |  |  |  |  |  |  |  |
|  | Living alone | 1.868 | **0.009** | -5.426 | 0.098 | -6.552 | **0.009** | -1.693 | 0.342 |
| Comorbidity | |  |  |  |  |  |  |  |  |
| No coexisting chronic disease | | -0.221 | 0.716 | 9.445 | **<0.001** | 4.289 | **0.044** | 0.996 | 0.508 |
| ≥1 coexisting chronic disease (reference) | |  |  |  |  |  |  |  |  |
| Antibiotics | |  |  |  |  |  |  |  |  |
| Yes (reference) | |  |  |  |  |  |  |  |  |
| No | | 1.041 | 0.096 | 1.683 | 0.555 | -2.420 | 0.272 | -0.199 | 0.898 |
| Not sure | | 1.513 | 0.202 | -6.925 | 0.200 | -4.162 | 0.319 | -4.107 | 0.161 |
| Hospitalization during the acute phase of infection | |  |  |  |  |  |  |  |  |
| Yes | | -0.356 | 0.649 | 0.168 | 0.963 | 3.587 | 0.193 | 2.487 | 0.199 |
| No (reference) | |  |  |  |  |  |  |  |  |

Note. EQ VAS = EQ visual analogue scale; MFI-20 = Multidimensional Fatigue Inventory 20-item version
p-values printed in bold indicate statistically significant values (p<0.05).

**Table C2.** Univariate logistic regression analyses for long-term post exertional malaise, depression and anxiety.

|  | | Post exertional malaise | | Depression | | Anxiety | |
| --- | --- | --- | --- | --- | --- | --- | --- |
|  | | Item 1 | | PHQ-2 | | GAD-2 | |
|  | | OR | p-value | OR | p-value | OR | p-value |
| Age at time of study | |  |  |  |  |  |  |
|  | <40 years old | 3.349 | 0.242 | 0.570 | 0.171 | 1.299 | 0.485 |
|  | 40 - <65 years old (reference) |  |  |  |  |  |  |
|  | ≥65 years old | 0.216 | **<0.001** | 0.378 | **0.003** | 0.445 | **0.021** |
| Gender | |  |  |  |  |  |  |
|  | Male | 1.087 | 0.842 | 2.121 | **0.002** | 1.192 | 0.489 |
|  | Female (reference) |  |  |  |  |  |  |
| Education | |  |  |  |  |  |  |
|  | Low | 2.100 | 0.184 | 1.393 | 0.192 | 1.983 | **0.011** |
|  | Middle (reference) |  |  |  |  |  |  |
|  | High | 0.402 | **0.029** | 0.819 | 0.435 | 0.671 | 0.165 |
| Living situation | |  |  |  |  |  |  |
|  | Not living alone (reference) |  |  |  |  |  |  |
|  | Living alone | 1.322 | 0.619 | 1.179 | 0.565 | 1.509 | 0.170 |
| Comorbidity | |  |  |  |  |  |  |
| No coexisting chronic disease | | 0.747 | 0.491 | 0.778 | 0.320 | 0.708 | 0.216 |
| ≥1 coexisting chronic disease (reference) | |  |  |  |  |  |  |
| Antibiotics | |  |  |  |  |  |  |
| Yes (reference) | |  |  |  |  |  |  |
| No | | 1.978 | 0.489 | 0.850 | 0.531 | 1.093 | 0.746 |
| Not sure | | - | - | 1.168 | 0.740 | 0.758 | 0.624 |
| Hospitalization during the acute phase of infection | |  |  |  |  |  |  |
| Yes | | 0.579 | 0.266 | 1.315 | 0.372 | 1.453 | 0.251 |
| No (reference) | |  |  |  |  |  |  |

Note. PEM item 1 = Marked, rapid physical and⁄or cognitive fatigability in response to exertion. PHQ-2 = Patient Health Questionnaire 2-item version, Generalized Anxiety Disorder 2-item version.
p-values printed in bold indicate statistically significant values (p<0.05).
